# The effects of insulin therapy on maternal blood pressure and weight in women with gestational diabetes mellitus

**DOI:** 10.1186/s12884-021-04066-z

**Published:** 2021-09-27

**Authors:** Tiange Sun, Fanhua Meng, Shufei Zang, Yue Li, Rui Zhang, Zhiyan Yu, Xinmei Huang, Fang Wang, Liwen Zhang, Jun Liu

**Affiliations:** 1 Department of Endocrinology, Shanghai Fifth People’s Hospital, Fudan University, 801 Heqin Road, 200240 Shanghai, China; 2 Department of Radiology, Shanghai Fifth People’s Hospital, Fudan University, 801 Heqin Road, 200240 Shanghai, China; 3 Department of Obstetrics and Gynecology, Shanghai Fifth People’s Hospital, Fudan University, 801 Heqin Road, 200240 Shanghai, China

**Keywords:** Gestational diabetes mellitus, Insulin therapy, Hypertension, Weight gain

## Abstract

**Background:**

Although insulin therapy achieves effective glycemic control, it may aggravate hyperinsulinemia. Nonetheless the benefits of insulin as first-line treatment for women with GDM are controversial. This work aimed to investigate the effect of insulin on maternal GDM.

**Methods:**

This retrospective cohort study recruited 708 women with GDM of whom 616 underwent lifestyle intervention and 92 were prescribed insulin therapy. Differences in variables between the two groups were analyzed by univariate analysis and multivariate analysis. Propensity score matching was used to control for age, pre-pregnancy BMI, time and BP at GDM diagnosis, and family history of diabetes and hypertension. Paired sample test was applied to evaluate the changes in BP after intervention in the two groups of women.

**Results:**

There was no significant difference in mode of delivery, newborn weight or incidence of macrosomia between women prescribed insulin and those who adopted lifestyle modifications. Insulin therapy was associated with a slight increase in maternal weight compared with the lifestyle intervention group and was attributed to short-term treatment (about 12 weeks). In addition, insulin therapy remarkably increased maternal blood pressure, an effect that persisted after matching age, pre-pregnancy BMI, time and BP at GDM diagnosis, and family history of diabetes and hypertension. Between commencing insulin therapy and delivery, systolic blood pressure significantly increased by 6mmHg (*P* = 0.015) and diastolic blood pressure by 9 mmHg (*P* < 0.001). Increase in BP was significantly higher in the insulin group compared with the lifestyle intervention group (*P* < 0.001). Logistic regression analysis with enter selection confirmed that insulin therapy was closely correlated with development of gestational hypertension (GH).

**Conclusions:**

This work suggested that short-term insulin therapy for GDM was associated with a slight increase in maternal weight but a significant risk of increasing maternal blood pressure.

## Introduction

### Background

Gestational diabetes mellitus (GDM) is defined as “diabetes diagnosed in the second or third trimester of pregnancy that was not clearly overt diabetes prior to gestation” [1]. With a greater prevalence of obesity and sedentary lifestyle, the prevalence of GDM is increasing globally. GDM is associated with a higher risk of serious complications for the mother (pre-eclampsia, caesarean section and development of type 2 diabetes) and the offspring (fetal macrosomia and childhood obesity) [2, 3–6].

Development of GDM is generally associated with overweight or obesity [7], and insulin resistance is the major pathophysiologic feature of women with GDM. To date, three classes of drugs have been recommended by the American Diabetes Association (ADA) for glycemic control in women with GDM. Insulin is first-line treatment. Metformin and glyburide should be used only as second-line treatment since both can cross the placenta to the fetus. Glyburide has been reported to be associated with a higher rate of neonatal hypoglycemia and macrosomia than insulin or metformin [8] although metformin may lead to nutrient restriction of glucose and amino acids to the fetus [9]. Exposure of the fetus to metformin may result in rapid growth after birth and a higher body mass index (BMI) by mid-childhood (5 to 9 years), effects associated with long-term metabolic consequences in later life such as obesity, type 2 diabetes, and cardiovascular disease [10]. Insulin is considered safe for the fetus since it does not cross the placenta to a measurable extent and is currently the first-line recommended treatment for GDM. Nonetheless the maternal effects of insulin therapy have not been studied. In addition, insulin may not improve the pathophysiologic features of women with GDM due to its risk of hyperinsulinemia which causes increased sodium reabsorption from the renal tubules, renin secretion, and sympathetic nervous activity [11].

This retrospective cohort study aimed to investigate the maternal and neonatal outcomes of insulin therapy. We established that insulin therapy for women with GDM was safe for the fetus, but that short term administration led to mild maternal weight gain and significantly increased BP.

### Research design and methods

This was a retrospective cohort study. From May 2013 to July 2019, we recruited all women with GDM at the Gestational Diabetes Mellitus Care Center of the Fifth People’s Hospital of Shanghai, Fudan University. The health card of all pregnant women was obtained from 10 to 12 weeks’ gestation and included information about age, last menstruation, method of conception, parity, obstetric history, family history of diabetes, previous history of GDM, and pre-pregnancy weight. Subsequently at the first visit, at the time of oral glucose tolerance testing (OGTT) and one week prior to delivery, blood pressure (BP), weight, blood count (Sysmex XN9000, Japan), and biochemistry results (Cobas 8000, Roche, Switzerland) were recorded. Blood tests were performed in the morning after an overnight fast of at least 8 h. BP was measured on two occasions 4 h apart. BMI was calculated as weight in kilograms divided by the square of the height in meters. Total gestational weight gain (GWG) and rate of weight gain during intervention was categorized according to the 2009 Institute of Medicine recommendations [12] as (1) inadequate weight gain; (2) adequate weight gain; (3) excessive weight gain.

Weighing scale was provided by Guangzhou GRG metrology and test CO. LTD, China (RGZ − 120). All women removed their shoes and heavy clothing prior to weight measurement. Blood pressure was measured using an automated sphygmomanometer (HEM − 7124) and recorded in both arms with the higher value being recorded. Normal weight women used a standard cuff (24 cm in length 12 cm in width) and overweight women an extended and widened cuff (64 cm in length 17 cm in width). Measurements were recorded with women seated in a chair with back support, their arm supported at heart level and after a minimum of 5 min rest. Weighing scale and automated sphygmomanometers were calibrated every 6 months and all measurements were taken by the same outpatient nurse using the same type of equipment. In the presence of raised BP or a diagnosis of GDM, routine obstetric examination was performed every 2 to 4 weeks in the outpatient clinic until 34 weeks’ gestation and thereafter every week.

Gestational hypertension (GH) was defined as a systolic blood pressure (SBP) ≥ 140 mmHg and/or a diastolic blood pressure (DBP) ≥ 90 mmHg on two occasions at least 4 h apart after 20 weeks of gestation in a woman with previously normal blood pressure [13].

All subjects with the exception of those diagnosed with overt diabetes or GDM in early pregnancy underwent routine screening for GDM at 24–28 weeks’ gestation according to a 75 g OGTT. A diagnosis of GDM was based on American Diabetes Association (ADA) diagnostic criteria.

The values for Homeostatic Model Assessment of Insulin Resistance (HOMA - IR) and Homeostatic Model Assessment of islet *β* cell function (HOMA - *β*) were determined from fasting plasma glucose and insulin concentration (Electrochemiluminescence, Cobas e602, Germany) [14]. Area under blood glucose (BG) curve was roughly calculated as $\frac{1}{2}\times \left(\text{f}\text{a}\text{s}\text{t}\text{i}\text{n}\text{g} \text{b}\text{l}\text{o}\text{o}\text{d} \text{g}\text{l}\text{u}\text{c}\text{o}\text{s}\text{e} \right($FBG) + 1 h BG) + $\frac{1}{2}\times$(1 h BG + 2 h BG).

Mothers with GDM were recommended to implement lifestyle modifications immediately on diagnosis of GDM and to continue until delivery. The time of GDM diagnosis was defined as the week of pregnancy in which changes were initiated. Women with GDM were advised to undertake a brisk 30 min walks at least three days per week along with dietary modifications and weight management. If glycemic control did not achieve the targets recommended by the Fifth International Workshop - Conference on GDM (Fasting glucose, 5.3 mmol/L or 1 h postprandial glucose, 7.8 mmol/L or 2 h postprandial glucose, 6.7 mmol/L) [15] after one week of lifestyle intervention, insulin therapy was commenced and continued along with lifestyle modifications until delivery according to ADA recommendations. Changes in weight and blood pressure were monitored from the time of GDM diagnosis until one week before delivery.

After delivery, data including gestational age at delivery, treatment protocol for lowering glycemia, birth weight, and gender of the neonate were recorded by medical staff.

**Fig. 1:**
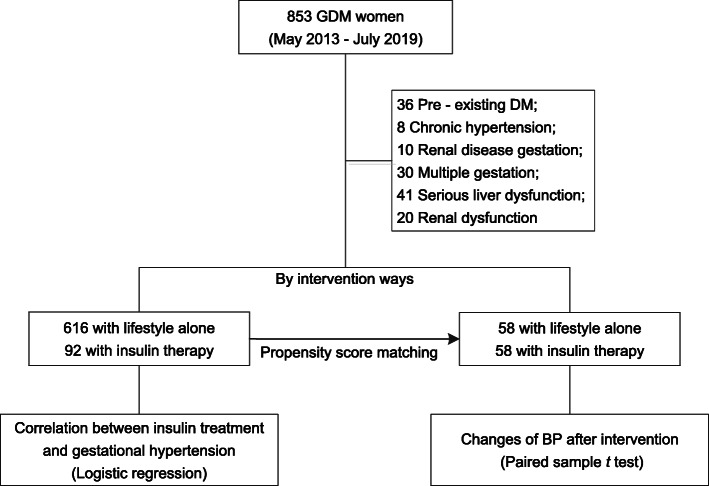
Flowchart of the study. GDM, gestational diabetes mellitus; GH, gestational hypertension

Women were excluded from the study for any of the following: ⑴ pre-existing DM; ⑵ chronic hypertension; ⑶ renal disease history; ⑷ multiple gestation; (5) serious liver dysfunction (alanine transaminase above 2.5 times upper limit) and renal dysfunction (estimated Glomerular Filtration Rate below 90 ml/min/1.73m^2^). Finally, 708 women with GDM were entered into the analysis. Retrospective analysis processes are shown in Fig. 1.

### Statistical analysis

Descriptive statistics for studied variables are presented as mean ± standard deviation (SD) for normally distributed variables, median (interquartile range (IQR)) for non-normally distributed variables, and frequency (percentage) for categorical variables. Student’s *t* - test or Mann-Whitney *U* test was used to identify difference in mean between groups. HOMA-IR and HOMA-*β* were log transformed previously for *t* tests. To eliminate the confounding effects on BP, a propensity score matching (PSM) method was employed to match variables of age, pre-pregnancy BMI, time (weeks) and BP at GDM diagnosis, family history of diabetes and hypertension. Matching tolerance was 0.02. Linear correlation of BP and other continuous variables was assessed by Pearson correlation analysis. Logistic regression analysis was performed with GH classified in a binary manner (presence/absence) as the dependent variable. Insulin therapy (categorized as present or absent) and other possible risk factors were entered into logistic regression analysis. Effect estimates are reported along with *OR* value, 95 % *CI* and *P* value. Changes to BP after intervention (from the time of GDM diagnosis to one week before delivery) were subjected to paired sample test. All analyses were performed using SPSS 24.0 (IBM SPSS lnc, Chicago, IL, USA). A two-tailed *P* value *<* 0.05 was considered statistically significant.

## Results

### Characteristics of subjects in lifestyle intervention group and insulin therapy group in full cohort

In women diagnosed with GDM, Treatment with insulin was added to the diet regimen after one week if fasting and postprandial blood glucose didn’t achieve the target. In this study, 92 women (12.9 %) required supplementary insulin therapy in addition to lifestyle modification and 616 women (87.1 %) required lifestyle modification alone. Table 1 illustrated mothers with insulin treatment were more likely to have family history of diabetes or lower HOMA - *β* (94.90 (52.05–259.19) vs. 295.85 (162.47–663.78), *P* < 0.001) comparing with those without. There were no significant differences in pre-pregnancy BMI, HOMA-IR, serum lipid parameters, or SBP and DBP at baseline between the two groups. One week before delivery, women treated with insulin had a much higher BMI (30.9 ± 4.5 vs. 28.4 ± 3.5 kg/m^2^, *P* = 0.020), SBP (131 ± 13 vs. 124 ± 13 mmHg, *P* = 0.005), DBP (80 ± 8 vs. 74 ± 9 mmHg, *P* = 0.007), and incidence of GH (31.0 % vs. 11.7 %, *P* = 0.001). Nonetheless the level of excessive weight gain showed no significant difference even though women prescribed insulin had a higher BMI than those adopting only lifestyle modifications (Table 1). There were statistically significant changes to DBP in women prescribed insulin therapy. Mean DBP increased from 70 ± 8 mmHg to 80 ± 8 mmHg, an increase of 9mmHg (95 % CI: 6–12 mmHg, *P* < 0.001), and SBP from 124 ± 11 mmHg to 131 ± 13 mmHg, an increase of 6 mmHg (95 % CI: 1–11 mmHg, *P* = 0.015). In comparison, in women with lifestyle intervention alone, mean SBP increased by 4mmHg (95 % CI: 2–6 mmHg, *P* < 0.001) and DBP by 5 mmHg (95 % CI: 4–7 mmHg, *P* < 0.001). Difference in changes to DBP (9 mmHg vs. 5 mmHg, *P* = 0.032), not SBP (6 mmHg vs. 4mmHg, *P* = 0.529) was significant between women prescribed insulin and those not. Finally, there was no difference between time of intervention for hyperglycemia (12.3 ± 1.6 vs. 12.6 ± 3.1 weeks, *P* = 0.628), glycemic control (FBG 4.39 ± 0.23 vs. 4.66 ± 0.33 mmol/L, *P* = 0.912; 1 h BG 7.14 ± 1.23 vs. 7.61 ± 1.09 mmol/L, *P* = 0.175; 2 h BG 6.98 ± 1.31 vs. 6.79 ± 1.22 mmol/L, *P* = 0.614 and HbA1c 5.2 ± 0.6 vs. 5.4 ± 0.4 %, *P* = 0.566 at delivery), delivery mode, preterm, newborn weight, or incidence of macrosomia between women with insulin and without (Table 1).

**Table 1:** Characteristics of women with GDM in lifestyle intervention group and insulin therapy group

	Full cohort			After PSM		
	Lifestyle intervention group	Insulin therapy group	*P*	Lifestyle intervention group	Insulin therapy group	*P*
* n*	616	92		58	58	
Age (years)	29.5 ± 5.0	30.7 ± 4.1	0.125	31.4 ± 6.4	30.7 ± 4.1	0.656
Pre-pregnancy BMI (kg/m^2^)	23.9 ± 4.0	24.8 ± 4.3	0.152	25.1 ± 1.9	24.6 ± 4.3	0.820
Family history of Hypertension	46 (10.0)	10 (11.6)	0.649	8 (13.7)	7 (12.1)	0.724
Family history of DM	9 (6.3)	8 (25.0)	***0.001 ***	4 (6.9)	5 (8.6)	0.624
At the first visit						
SBP (mmHg)	118 ± 11	119 ± 12	0.627	121 ± 10	117 ± 12	0.328
DBP(mmHg)	71 ± 8	70 ± 7	0.630	69 ± 8	70 ± 7	0.382
Creatin (μmol/L)	39.65 ± 6.37	40.71 ± 5.82	0.668	53.00 ± 19.03	40.71 ± 5.82	0.326
UA (mmol/L)	225.65 ± 43.08	236.14 ± 90.23	0.660	266.33 ± 29.73	236.14 ± 90.23	0.597
eGFR (ml/min/1.73m^2^)	180.6 ± 31.7	171.2 ± 24.8	0.443	145.4 ± 39.7	170.2 ± 24.8	0.281
ALT (units/L)	19.0 (13.0 - 34.0)	23.0 (15.0 - 80.0)	0.257	16.0 (11.0 - 70.0)	23.0 (15.0 - 80.0)	0.667
AST (units/L)	18.5 (15.0 - 22.5)	17.0 (16.0- 20.0)	0.727	26.0 (14.0 - 33.0)	17.0 (16.0 - 20.0)	0.517
At performing OGTT						
OGTT time (week)	25.8 ± 2.9	24.3 ± 4.8	***0.042 ***	26.4 ± 4.8	24.3 ± 4.8	0.185
BMI (kg/m^2^)	26.5 ± 3.7	27.6 ± 4.4	0.213	27.3 ± 2.0	27.6 ± 4.4	0.802
SBP (mmHg)	120 ± 12	124 ± 11	0.083	126 ± 11	125 ± 11	0.693
DBP (mmHg)	69 ± 9	70 ± 8	0.525	74 ± 8	71 ± 10	0.241
FBG (mmol/L)	4.70 ± 0.73	5.61 ± 1.48	***< 0.001***	3.75 ± 0.21	5.60 ± 1.48	*< 0.001*
1 h BG (mmol/L)	9.64 ± 1.63	11.32 ± 2.20	***< 0.001***	9.55 ± 1.35	11.32 ± 2.20	***0.011 ***
2 h BG (mmol/L)	8.29 ± 1.55	9.61 ± 2.49	***0.002 ***	8.67 ± 0.87	9.61 ± 2.48	***0.047 ***
HbA1c (%)	5.2 ± 0.4	5.6 ± 0.6	***< 0.001***	5.2 ± 0.3	5.5 ± 0.6	***0.027 ***
HbA1c (mmol/mol)	33	38		33	37	
Area under BG curve	16.14 ± 1.93	18.86 ± 3.80	***< 0.001***	16.02 ± 1.14	18.86 ± 3.80	***0.025 ***
HOMA - IR^*^	2.54 (1.66 - 3.45)	2.30 (1.66 - 3.63)	0.716	1.77 (1.09 - 2.95)	3.00 (1.53 - 3.63)	0.179
HOMA - β^*^	295.85 (162.47 - 663.78)	94.90 (52.05 - 259.19)	***< 0.001***	331.28 (80.94 - 823.42)	94.90 (52.05 - 259.19)	***< 0.001***
CH (mmol/L)	5.50 ± 1.26	5.47 ± 1.30	0.896	5.56 ± 1.44	5.47 ± 1.30	0.832
TG (mmol/L)	3.18 ± 1.76	3.05 ± 1.82	0.667	2.93 ± 1.30	3.05 ± 1.82	0.845
HDL (mmol/L)	1.73 ± 0.45	1.67 ± 0.42	0.521	1.58 ± 0.21	1.67 ± 0.42	0.481
LDL (mmol/L)	2.93 ± 1.04	2.96 ± 1.02	0.897	3.12 ± 0.97	2.95 ± 1.02	0.651
One week before delivery						
Delivery time	38.6 ± 1.5	39.1 ± 1.3	0.170	39.0 ± 0.9	39.2 ± 1.3	0.664
intervention time frame (weeks)	12.6 ± 3.1	12.3 ± 1.6	0.628	12.6 ± 4.5	12.3 ± 1.6	0.809
Insulin dose	NA	39 (27 - 56)		NA	39 (27 - 56)	
BMI (kg/m^2^)	28.4 ± 3.5	30.9 ± 4.5	***0.020 ***	28.8 ± 2.2	30.4 ± 4.7	***0.030 ***
SBP (mmHg)	124 ± 13	131 ± 13	***0.005 ***	124 ± 9	131 ± 12	***0.043 ***
DBP (mmHg)	74 ± 9	80 ± 8	***0.007 ***	71 ± 10	80 ± 11	*0.011 *
FBG (mmol/L)	4.66 ± 0.33	4.39 ± 0.23	0.912	4.51 ± 0.20	4.82 ± 0.42	0.711
1 h BG (mmol/L)	7.61 ± 1.09	7.14 ± 1.23	0.175	7.39 ± 1.47	7.50 ± 1.22	0.845
2 h BG (mmol/L)	6.79 ± 1.22	6.98 ± 1.31	0.614	7.24 ± 1.54	6.73 ± 0.75	0.306
HbA1c (%)	5.4 ± 0.4	5.2 ± 0.6	0.566	5.3 ± 0.4	5.4 ± 1.3	0.699
HbA1c (mmol/mol)	36	33		34	37	
Total GWG (kg)			0.068			0.223
Adequate	225 (42.1)	12 (16.7)		21 (38.2)	9 (16.7)	
Inadequate	171 (31.3)	24 (33.3)		16 (29.1)	18 (33.3)	
Exceesive	150 (27.5)	36 (50.0)		18 (32.7)	27 (50.0)	
Rate of weight gain after OGTT (kg week^-1^)			0.345			0.563
Adequate	84 (28.6)	12 (11.8)		18 (32.7)	16 (30.2)	
Inadequate	72 (24.5)	15 (29.4)		14 (25.5)	6 (11.3)	
Exceesive	138 (46.9)	60 (58.8)		23 (41.8)	31 (58.5)	
Gestational hypertension	72 (11.7)	20 (31.0)	*0.001 *	12 (17.2)	26 (38.3)	***0.028 ***
Caesarean section	280 (68.3)	46 (63.9)	0.602	28 (48.3)	32 (56.5)	0.449
Fetal sex (female)	310 (49.1)	22 (37.9)	0.260	28 (50.9)	21 (37.5)	0.321
Preterm	14 (4.7)	2 (6.3)	0.779	3 (5.5)	3 (5.3)	0.932
Newborn weight (g)	3484.5 ± 488.7	3592.1 ± 638.5	0.260	3607.5 ± 392.1	3592.1 ± 638.5	0.938
Macrosomia	62 (14.3)	10 (15.2)	0.796	9 (16.3)	8 (15.1)	0.901

Data are mean ± SD, median (interquartile range) or n (%), Log transformed for t test*

*GDM* gestational diabetes mellitus; *Cr* creatinine; *UA* uric acid; *ALT* alanine aminotransferase; *AST* aspartate aminotransferase; *GWG* gestational weight gain; *SBP* systolic blood pressure; *DBP* diastolic blood pressure; *eGFR* estimated glomerular filtration rate; *PSM* propensity score matching

PSM to even the variables of age, pre-pregnancy BMI, time (weeks) and BP at GDM diagnosis, family history of diabetes and hypertension, match tolerance was 0.02

### Comparison of parameters between women with lifestyle intervention alone and those prescribed insulin therapy after PSM

**Fig. 2:**
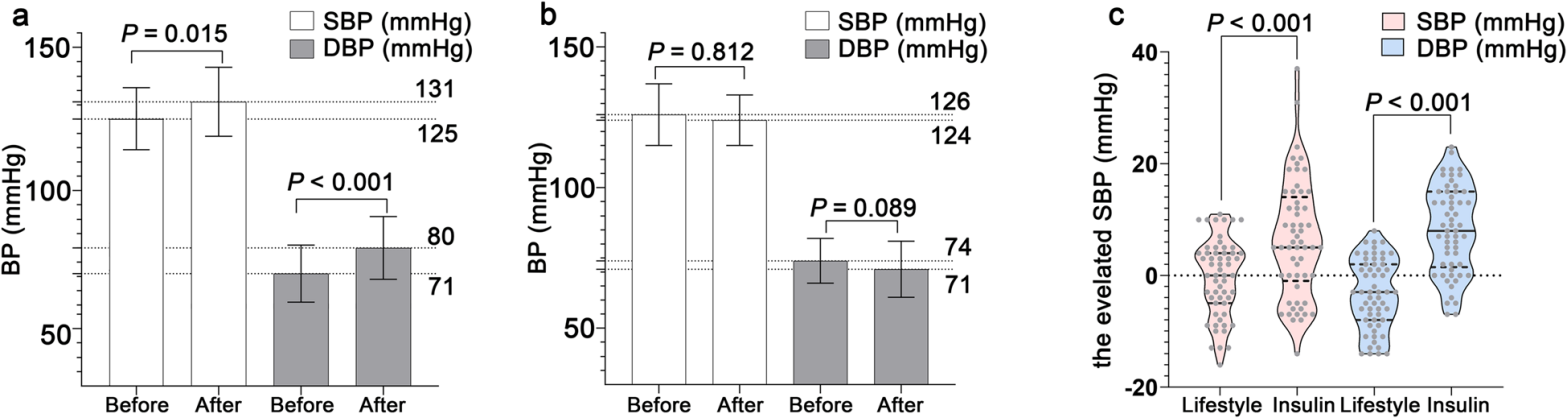
Comparison of changes to BP in GDM women after propensity score matching. Figure **a** shows changes to BP in women with insulin therapy; Figure **b** shows changes to BP in women with lifestyle intervention alone; Figure **c** shows comparison of the elevated BP between women with lifestyle changes alone and those with insulin therapy. GDM, gestational diabetes mellitus; SBP, systolic blood pressure; DBP, diastolic blood pressure

To eliminate confounding effects on BP, a PSM method was employed to match variables of age, pre-pregnancy BMI, time (weeks) and BP at GDM diagnosis, family history of diabetes and hypertension. After matching, there were no significant differences in glycemic control, intervention time frame for glycemic control, the ratio of excessive weight gain, delivery mode, newborn weight or incidence of macrosomia between women with different intervention ways. Nonetheless there remained a higher incidence of GH (38.3 % vs. 17.2 %, *P* = 0.028), and increased BMI (30.4 ± 4.7 vs. 28.8 ± 2.2 kg/m^2^*P* = 0.030), SBP (131 ± 12 vs. 124 ± 9 mmHg, *P* = 0.043) and DBP (80 ± 11 vs. 71 ± 10 mmHg, *P* = 0.011) as time of delivery approached in women with insulin therapy compared with those with lifestyle intervention (Table 1). Paired sample *t* test revealed that changes to SBP and DBP in women prescribed insulin were statistically significant. SBP increased from 125 ± 11 to 131 ± 12 mmHg, an increase of 6mmHg (95 % CI: 1–12 mmHg, *P* = 0.015), and DBP from 71 ± 10 to 80 ± 11 mmHg, an increase of 9 mmHg (95 % CI: 6–12 mmHg, *P* < 0.001) (Fig. 2a). On the contrary, in women who adopted only lifestyle modifications, changes to SBP (*P* = 0.812) and DBP (*P* = 0.089) were not significant (Fig. 2b). In addition, there were significant differences in the elevated SBP (*P* < 0.001) and DBP between the two groups (*P* < 0.001) (Fig. 2c).

### Association of blood pressure with metabolic parameters (*n* = 503)

As shown in Table 2, BP in the week of performing OGTT was closely associated with HOMA-IR (*r* = 0.25, *P* = 0.026) and pre-pregnancy BMI (*r* = 0.21, *P* = 0.039). SBP at the time of delivery had a significant positive correlation with 1 h BG (*r* = 0.24, *P* = 0.010), HbA1c (*r* = 0.25, *P* = 0.010), area under BG curve (*r* = 0.35, *P* < 0.001), BMI (*r* = 0.39, *P* < 0.001), and total GWG (*r* = 0.22, *P* = 0.029).

**Table 2:** The correlation analysis of blood pressure and other variables (*n* = 503)

	At OGTT				Approaching delivery			
	SBP	*P*	DBP	*P*	SBP	*P*	DBP	*P*
Age (years)	-0.05	0.620	-0.16	0.101	-0.02	0.840	-0.10	0.321
Pre-pregnancy BMI (kg/m2)Pre-pregnancy BMI (kg/m2)	0.21	***0.039 ***	0.23	***0.019***	0.21	***0.030 ***	0.15	0.140
At performing OGTT								
BMI (kg/m2) BMI (kg/m2)	0.24	***0.025 ***	0.21	0.059	0.35	***< 0.001***	0.33	***0.002 ***
FBG (mmol/L)	0.14	0.172	-0.08	0.437	0.09	0.390	0.16	0.126
1 h BG (mmol/L)	0.03	0.777	-0.07	0.455	0.24	***0.010 ***	0.15	0.124
2 h BG (mmol/L)	-0.03	0.761	-0.08	0.41	0.18	0.070	0.11	0.253
HbA1c (%)	0.17	0.083	0.10	0.329	0.25	***0.010 ***	0.14	0.178
Area under BG curve	0.07	0.498	-0.05	0.628	0.35	***< 0.001***	0.23	***0.028 ***
HOMA - IR* HOMA - IR*	0.25	***0.026 ***	0.16	0.162	0.07	0.530	0.11	0.314
HOMA - β* HOMA - β*	-0.03	0.788	0.17	0.156	-0.19	0.120	-0.18	0.132
CH (mmol/L)	-0.01	0.959	-0.08	0.421	0.04	0.690	0.01	0.896
TG (mmol/L)	0.09	0.366	0.16	0.114	0.18	0.070	0.15	0.126
HDL (mmol/L)	-0.12	0.246	-0.04	0.685	-0.02	0.840	-0.04	0.677
LDL (mmol/L)	0.01	0.947	-0.10	0.336	0.02	0.850	0.00	0.979
One week before delivery								
BMI (kg/m2)	NA	NA	NA	NA	0.39	***< 0.001***	0.29	***0.030 ***
Total GWG (kg)	NA	NA	NA	NA	0.22	***0.029 ***	0.12	0.224
Rate of weight gain after intervention (kg week-1)	NA	NA	NA	NA	0.07	0.950	0.036	0.751

Log transformed for the correlation analysis*

*GWG* gestational weight gain; *SBP* systolic blood pressure; *DBP* diastolic blood pressure

### Insulin therapy was closely associated with development of GH (*n* = 512)

Logistic regression analysis with enter selection showed that insulin therapy was closely correlated with development of GH (*OR* = 6.33; 95 %CI, 1.17 to 34.09 vs. the lifestyle intervention, *P* = 0.032) corrected by history of hypertension, 2 h BG and total GWG. The same result was obtained when rate of weight gain during intervention instead of total GWG was entered into the model (*OR* = 6.65; 95 %CI, 1.14 to 38.65 vs. the lifestyle intervention, *P* = 0.035) (Table 3).

**Table 3:** Logistic regression analysis with enter selection for presence/absence GH (*n* = 512)

	*B*	*OR*	*95% CI*	*P*
Insulin therapy	1.85	6.33	1.17 - 34.09	***0.032***
History of hypertension	3.10	22.4	2.66 -188.96	***0.004***
2h BG (mmol/L)	0.63	1.88	1.10 - 3.21	***0.020 ***
Total GWG (kg)				
Adequate	reference			
Inadequate	-1.24	0.29	0.04 - 2.18	0.228
Excessive	2.07	7.89	1.63 – 38.15	***0.010 ***
Insulin therapy	1.89	6.65	1.14 - 38.65	***0.035***
History of hypertension	4.18	65.13	3.81 - 703.54	***0.004***
2h BG (mmol/L)	0.50	1.65	0.91 – 2.99	0.099
The rate of weight gain during intervention (kg week^-1^)				
Adequate	reference			
Inadequate	0.02	1.02	0.06 – 18.50	0.992
Excessive	2.87	17.56	1.05 – 192.42	**0.046**

*GH* gestational hypertension; *BG* blood glucose; *GWG* gestational weight gain

## Discussion

Insulin is recommended as first-line treatment for hyperglycemia in GDM by the ADA and many other associations. In this retrospective cohort study, we assessed the effect of insulin therapy on mothers with GDM and their fetus. Insulin therapy for about 12 weeks had a mild effect on maternal weight although it markedly increased BP. To avoid confounding, a PSM method was applied and revealed that the effect of insulin on maternal BP persisted.

Previous studies have evaluated the effects of insulin versus oral anti-diabetic drugs for treatment of GDM. Compared with oral anti-diabetic drugs, insulin has been associated with higher weight gain in GDM women [16, 17]. Nonetheless in infants, no significant differences in risk of perinatal death, being born large-for-gestational age [18, 19] or serious neonatal outcomes [20, 21] have been reported. Insulin resistance/hyperinsulinemia is largely attributed to obesity, a vital physiological character of GDM. Although insulin therapy achieves effective glycemic control, it may aggravate hyperinsulinemia.

Studies that have compared the effects on mothers with GDM and/or their fetus of insulin and lifestyle intervention showed no difference in weight gain between the two interventions. Our study retrospectively analyzed the effects of insulin therapy on maternal and neonatal outcomes and consistent with other studies found no difference in delivery mode, preterm delivery, or being born with macrosomia [22, 23].

It is generally considered that insulin therapy may induce weight gain in diabetic patients [24, 25]. Physiological fluctuations in insulin play a critical role in the balance between energy storage and energy consumption. Insulin acts as an anabolic hormone to reduce lipolysis and protein catabolism and promote lipogenesis and protein formation. Studies in humans and mice have postulated that over-replacement of insulin in patients with diabetes produces a general anabolic effect that leads to increased fat accumulation and weight gain [24]. Another mechanism proposed is that exogenous insulin first enters the peripheral tissues, such as adipose tissue and muscle tissue, resulting in increased fat synthesis. This leads to a cluster of ectopic fat accumulation and insulin resistance. In our cohort study, the length of insulin treatment was only about 12 weeks in women with GDM. That didn’t lead to excessive weight gain in such women, however they had a higher BMI before delivery than those adopting lifestyle modifications alone. The results hinted that short-term use of insulin results in a mild increase in weight, but if administered for longer than 12 weeks the adverse effect might be aggravated.

Niromanesh et al. [26] reported that pregnant women prescribed insulin therapy had higher BP and weight gain than those given metformin. Our study showed that insulin therapy increased BP, and BP was positively associated with BMI and HOMA-IR. Wang F et al. [27] demonstrated that insulin resistance or elevated fasting insulin concentration was independently associated with an exacerbated risk of hypertension in the general population. Hyperinsulinemia/insulin resistance may induce sodium reabsorption by the distal nephron segments [28], resulting in increased release of angiotensin II, the main effector peptide of the renin-angiotensin system [29], and enhanced sympathetic activity, vascular resistance [30, 31], and endothelial dysfunction [29]. Ana et al. [32] aimed to elucidate the mechanism by which hyperinsulinemia increases BP during pregnancy using euglycemic hyperinsulinemia and normal pregnant Sprague-Dawley rats. The results demonstrated that sustained euglycemic hyperinsulinemia could raise blood pressure in pregnancy, independent of changes in glycemia. Wang et al. [33] reported similar results in Sprague-Dawley rat models in which hyperinsulinemia rather than insulin resistance played an important role in blood pressure elevation. The reduction of urinary sodium excretion also appeared to be an important mediator to link hyperinsulinemia and blood pressure. Similar results were evident in our study. It is established that development of GDM shares the same risk factors as GH. To avoid bias, we matched variables that could affect BP prior to intervention (there was similar insulin resistance between two groups), and found no significant difference in glycemic control between the two groups although SBP and DBP remained higher in women prescribed insulin. The results also imply the effect of insulin of increasing BP independently of changes in glycemia and insulin resistance.

Insulin is currently considered first-line therapy for glycemic control in pregnant women. Nonetheless it may not be the best option for GDM since it does not alter the pathophysiology. New medicine needed to be developed or found which can improve the pathophysiology of GDM and be safety for mothers and fetus.

There were some limitations of this study. All subjects were derived from one center and this may have led to biased results. Expanding the sample size or using a reliable rodent model of GDM is needed to delineate the effect and mechanism of insulin therapy on pregnancy.

## Conclusions

In summary, our study indicated that insulin therapy was safe for the fetus, and had a slight effect on maternal weight with short-term use but remarkably increased maternal BP. When insulin is used to control glycemia, clinicians should take care to monitor maternal BP and weight gain if long-term use is being considered.

## Data Availability

All data and material used and/or analyzed during the current study are not publicly available but are available from the corresponding author on reasonable request.

## Abbreviations

GDM: Gestational diabetes mellitus
ADA: American Diabetes Association
BMI: Body mass index
GH: Gestational hypertension
OGTT: Oral glucose tolerance test
BP: Blood pressure
GWG: Total gestational weight gain
SBP: Systolic blood pressure
DBP: Diastolic blood pressure
HOMA - IR: Homeostatic Model Assessment of Insulin Resistance
HOMA - *β*: Homeostatic Model Assessment of Insulin Sensitivity
BG: Blood glucose
